# Correlation of death anxiety with coping strategies among Palestinian women with breast cancer: a cross-sectional study

**DOI:** 10.3389/fpubh.2024.1420306

**Published:** 2024-06-10

**Authors:** Muna Ahmead, Feda Shehadah, Issa Abuiram

**Affiliations:** Faculty of Public Health, Al-Quds University, Jerusalem, Palestine

**Keywords:** breast cancer, fear, death anxiety, women, coping strategies, Palestine

## Abstract

**Background:**

Death anxiety and maladaptive coping accompany breast cancer diagnoses. The coping mechanisms and death anxiety among Palestinian patients with breast cancer have not been studied.

**Aim:**

To assess the prevalence of death anxiety and its relationship with coping strategies among Palestinian women with breast cancer who are treated in Beit Jala Governmental Hospital in Bethlehem.

**Method:**

A cross-sectional design was used, and 214 breast cancer patients who visited the Beit Jala Governmental Hospital in Bethlehem were recruited. Templer's Death Anxiety Scale and the Brief COPE Scale were used. To investigate the relationship between coping strategies and death anxiety, frequency, percentages, chi-square tests, and Pearson's correlation tests were utilized.

**Results:**

The results indicated that 58.40% of the patients experienced death anxiety. The participants who used positive reframing (adjusted odds ratio (AOR) = 1.487, *p* = < 0.026), self-blame (AOR = 1.309, *p* = < 0.023), and religion (AOR = 1.260, *p* = < 0.031) as coping mechanisms were more likely to experience death anxiety. Conversely, the participants who adopted substance use (AOR = 0.657, *p* < 0.005) and active coping (AOR = 0.629, *p* < 0.007) as coping strategies had a lower likelihood of experiencing death anxiety.

**Conclusion:**

The study revealed that breast cancer patients tended to use a combination of functional and emotional coping strategies and that a significant proportion of these patients (58.4%) experienced symptoms of death anxiety. This study emphasizes the significance of screening for death anxiety and understanding the coping strategies utilized by the patients. Gaining this understanding will assist in identifying patients who need more guidance and support.

## 1 Introduction

Breast cancer is the most frequently diagnosed cancer in women. According to the WHO, the global breast cancer incidence in 2022 was 2.3 million, with 670,000 deaths (1). Cancer typically elicits psychological reactions characterized by traumatic stress (2) and requires treatment with extreme caution due to the negative connotation associated with the term “cancer” (2, 3). Patients who are diagnosed with cancer experience profoundly intense psychological reactions, including a sense of impending death. As a result, death anxiety is widely regarded as the most important psychological factor affecting cancer management. Death anxiety is the emotional reaction that individuals exhibit when they encounter negative thoughts associated with death (4). It can harm cancer patients' psychological wellbeing, potentially leading to mental disorders and reduced overall quality of life (5, 6). Furthermore, death anxiety has a significant effect on cancer treatment and eradication (7). Cancer patients reported moderate levels of death anxiety, with women diagnosed with breast cancer reporting higher levels (8). According to Masror Roudsary et al.'s (9) study in Iran, 79% of patients with breast cancer have a fear of death. Furthermore, a cross-sectional study conducted in Pakistan on 80 patients with breast cancer revealed that 13.8%, 51.2%, 27.5%, and 7.5% of the patients had low, medium, high, and very high levels of death anxiety, respectively (10).

A fear of death among people arises from various reasons, including identity loss, anxiety about life after death, pain and distress, and loved ones' wellbeing. Fear of death affects patients' prognoses, leading to treatment skepticism or discontinuation (11). Many studies have explored cancer patients and their families' fear of death (3, 12–15). However, it is unclear what early-stage patients experience in terms of fear of death because most published research focuses on the terminally ill (16). Shakeri et al. (3) found that death anxiety scores in patients with breast cancer was 67.5%. Women harbored apprehensions about mortality, exacerbation of their illness, loss of their future, and dependence on other people (3).

Women diagnosed with breast cancer adopt coping strategies to navigate the complex and multifaceted challenges that arise from the psychological, social, and spiritual aspects of their new life circumstances (17). They must adjust to the changes that happen every day to lessen their pain and improve their quality of life (18). Coping is the mindset and actions adopted to maintain emotional health during cancer complications (19). Researchers found that patients with critical and terminal illnesses who use coping mechanisms live longer and increase life satisfaction (20). Emotion-focused coping manages strong emotions, while problem-focused coping addresses the source of distress (21). Patients with cancer often resort to denial, alcohol or drugs, venting, self-distraction, and behavioral disengagement; these strategies make them less adaptive (17). When cancer is diagnosed, these coping strategies increase the risk of depression and a decline in self-esteem (22). Religion-based problem-solving and coping methods are more adaptive and reduce depressive symptoms (23). However, there is a lack of quantitative studies that assess coping strategies among Palestinian women diagnosed with breast cancer. According to a qualitative study, Palestinian women with breast cancer most commonly rely on social support and religion as coping strategies (24). Pre-existing coping styles, as well as supportive relationships with others, have an impact on an individual's ability to effectively cope with illness. In addition, the perception of cancer as a threat appears to promote active coping behavior (2). Conversely, cancer patients who perceive their illness as long-term, emotionally burdensome, and with more negative consequences are more likely to employ passive coping strategies (25).

Throughout their cancer treatment, many breast cancer patients suffer from various mental health issues, including but not limited to anxiety, depression, cognitive impairment, distress, poor body image, and sexual dysfunction (26). In addition, stressful events such as accepting a diagnosis, beginning treatment, learning about the prognosis, addressing side effects, preparing for a relapse, and facing an unknown future can lead to mental instability and mood disorders such as depression and anxiety (27). Studies revealed that cognitive behavioral therapy, an intervention to improve coping and communication, mindfulness-based stress reduction, and supportive psychotherapy are all potential treatments that can effectively address the psychosocial problems faced by cancer patients (27).

In Palestine, breast cancer has perpetually been highly prevalent. Breast cancer incidence was 934 in 2022, with 18.5 cases per 100,000 people and 37.4 per 100,000 women. Cancer deaths among women were 45% (28). Palestinian cancer patients may struggle with infertility, changing social roles, body image, sexual issues, and strained relationships (29, 30). Many studies have found that Arab women may hide breast cancer from friends, family, and neighbors due to social stigma (29, 30). Moreover, Arab women perceive cancer as a punishment or a divine test. The concerns center on the stigma associated with breast examination, the potential reaction of the spouse upon discovering a lump, and the impact of cultural and religious modesty (30). This may exacerbate their psychological distress and anxiety about mortality, influencing breast cancer detection and prognosis (29, 30). According to Madkhali et al. (31), Arab women with breast cancer frequently seek medical attention only at the advanced stage of the disease, with large breast lumps that are difficult to manage at home and cause intense pain.

Despite the high prevalence of breast cancer among Arab and Palestinian women, there has been little research into the relationship between specific medical conditions such as breast cancer and death anxiety. Furthermore, most previous research on this topic has focused on students (32–34).

To our knowledge, no study has examined coping mechanisms and death anxiety in Palestinian or Arab breast cancer patients. In addition, Palestinian general hospitals lack qualified mental health professionals, which may increase death anxiety among breast cancer patients due to misdiagnosis and inadequate treatment. Therefore, this study aimed to assess the prevalence of death anxiety in Palestinian women diagnosed with breast cancer. Furthermore, it sought to assess the relationship between death anxiety and sociodemographic and psychological factors along with coping strategies. Finally, it explored the factors that predict the development of death anxiety among women with breast cancer. The results of the study may help healthcare practitioners in providing effective treatment for women with breast cancer.

## 2 Materials and methods

This study examined death anxiety and coping strategies among Palestinian breast cancer patients at Beit Jala Governmental Hospital in Bethlehem, Palestine. A cross-sectional study was conducted using self-reported questionnaires. Participants included Palestinian women with breast cancer treated at the Beit Jala Governmental Hospital, the only government hospital in Bethlehem and a referral center for Palestinian cancer patients requiring chemotherapy. The study focused on women aged 18 and above with breast cancer, typically in stage three or four, treated in the daycare center and outpatient clinics of the hospital. Women hospitalized for breast cancer, lung cancer, or colon cancer were excluded from the study. Due to the unavailability of official statistics on the number of breast cancer patients treated at the hospital, patients with breast cancer treated from April 2016 to January 2017 were recruited using a convenience sampling method. A total of 214 women agreed to participate. The researchers distributed questionnaires to participants in the daycare center and outpatient clinics during their hospital visits.

### 2.1 Measures

The data were collected using a questionnaire comprising three sections and a total of 53 questions. The first section included sociodemographic factors (i.e., age, place of residence, marital status, educational level, and economic status) and psychological variables (i.e., onset of breast cancer, whether seeking psychological treatment, psychological treatment obstacles, whether receiving psychological support, and source of psychological support).

The second section used *Templer's Death Anxiety Scale (Templer DAS)* to assess death anxiety (35). The scale comprises 15 questions that assess absolute death anxiety, fear of patience and pain, death-related thoughts, passing of time and short life, and fear of future. Each item has two possible answers, yes or no, with 1 and 0 values, respectively, and the true response indicates participants' anxiety. The Templer DAS scores range from 0 (no death anxiety) to 15 (highest death anxiety), with a cutoff score of 6. A class interval of 0–6 indicates no death anxiety, 7–8 indicates an average level of death anxiety, and 9–15 indicates a high level of death anxiety. Studies categorize death anxiety as low (0–6) or high (7–15) (36).

The third section had *the Brief COPE scale*, developed by Carver and comprising 28 questions (37). It is one of the most frequently used self-reporting tools for measuring coping responses. Both cognitive and behavioral strategies of coping are included. For each category, respondents indicate whether they have used a coping response on a four-point Likert scale, where 1 indicates “I have not been doing this at all,” 2 indicates “I have been doing this a little bit,” 3 indicates “I have been doing this an average amount,” and 4 indicates “I have been doing this a lot.” The higher the score, the greater are the coping strategies used by the respondents. In addition, the coping dimensions are divided into two major categories: problem-focused strategies (i.e., active coping, planning, and use of instrumental support) and emotion-focused strategies (i.e., positive reframing, acceptance, religion, use of emotional support, and denial).

As the scale was not previously evaluated among Palestinian cancer patients, a committee of three mental health specialists evaluated it for cultural appropriateness; no changes were required. A certified medical translator reverse-translated the scale from Arabic to English after the research team translated it. The accuracy of the translation was verified by comparing the original English questionnaire with the back-translated version. A pilot test was conducted by administering the tool to 15 patients with breast cancer for verifying language clarity. Cronbach's alpha values of 0.753 for the Templer DAS and 0.73 for the Brief COPE scale indicate good reliability.

### 2.2 Statistical analysis

The data were analyzed using the statistical package for social sciences (SPSS) version 18.0. The relationships of sociodemographic variables and psychological factors with the Templer DAS were analyzed using frequency, percentages, and the chi-square test. Pearson's correlation coefficient (r) was employed to examine the relationships between coping strategies and death anxiety. In addition, a multivariate regression analysis was performed, and the results were obtained as an adjusted odds ratio (AOR) with a 95% confidence interval (CI). A *p* < 0.05 was considered a significant association.

### 2.3 Ethical approval and consent to participate

This study followed the Helsinki Declaration for all methods and was ethically approved by the Faculty of Public Health/ Community Mental Health Ethical Research Committee at Al Quds University (Ref No. FPH: 1-4-2016). Subsequently, a formal letter explaining the study's purpose and the questionnaire were sent to the Palestinian Ministry of Health and Beit-Jala Governmental Hospital administration for permission. Participants received an information sheet about the study's purpose and were informed about the option to not participate in the study. All participants gave written informed consent and were assured confidentiality and privacy.

## 3 Results

A total of 214 participants agreed to participate in the study. According to Table 1, 36% (*n* = 76) of the participants were aged between 31 and 40 years, while 46% (*n* = 99) lived in cities. Furthermore, 69.7% of the participants (*n* = 149) were married, and 46.3% (*n* = 99) earned 601–900 US$ per month. In addition, 36% (*n* = 77) of the participants reported that their cancer began less than a year ago.

**Table 1:** Sociodemographic variables of the participants and onset of breast cancer.

		**n**	**%**
**Age (Years)**	18–30	22	10%
	31–40	76	36%
	41–50	53	25%
	51+	63	29%
**Place of residence**	City	99	46%
	Village	80	38%
	Camp	35	16%
**Marital status**	Married	149	69.7%
	Widow	38	17.8%
	Single	14	6.5%
	Divorced	13	6 %
**Education level**	University degree	62	29%
	Secondary (10–12 years)	57	26%
	Preparatory (6–9 years)	33	15%
	Elementary (1–6 years)	30	15%
	Illiterate	32	15%
**Economic status**	No income	15	7.0%
	Less than 300 US$	19	8.9%
	301 US$-600 US$	37	17.2
	601 US$-900 US$	99	46.3%
	≥ 901 US$	44	20.6%
**Onset of breast cancer**	Less than 1 year	77	36%
	1–2 years	74	34.6%
	3–5 years	32	15%
	6+ years	31	14.4%

As shown in Table 2, 30.8% (*n* = 66) of the participants reported that they suffered from psychological problems such as depression and anxiety. The participants were discouraged from seeking therapy because 12.1% (*n* = 26) thought it was ineffective, 40.1% (*n* = 86) cited their family's financial situation, and 8.8% (*n* = 19) were concerned about the stigma associated with seeking therapy. When asked if they had received any social support, 11.2% (*n* = 24) answered no and only 9.3% (*n* = 20) said that they received support from their husbands.

**Table 2:** Psychological variables of the participants.

		***n***	**%**
Did you suffer from any psychological problems such as depression and anxiety?	Yes	66	30.8%
	No	148	69.2%
Did you seek any psychological treatment?	Yes	18	8.4%
	No	196	91.6%
Psychological treatment obstacles	Psychotherapy not effective	26	12.1%
	Economic situation	86	40.1%
	Stigma	19	8.8%
	Not aware of the places that offer psychological services	51	23.8%
	Religious reasons	8	4%
	I do not want to answer	24	11.2%
Did you receive any social support?	Yes	190	88.8%
	No	24	11.2%
Source of social support	Father	82	38.3%
	Sister	25	11.6%
	Friend	19	9 %
	Mother	19	9%
	Husband	20	9.3%
	Brother	15	7%
	Others	3	1.4%
	None	31	14.4%

As shown in Table 3, the mean Templer DAS scores for death anxiety among breast cancer patients were moderate (mea*n* = 7.0, standard deviation (SD) = 1.6). Furthermore, 39.8% of the participants had an average level of death anxiety, 41.6% had an absence of death anxiety, and 18.6% had high levels of death anxiety.

**Table 3:** Mean and SD of death anxiety categories.

**Death anxiety categories**	***n***	**%**
Absence of death anxiety (0–6)	89	41.6%
Average level of anxiety (7–8)	85	39.8%
High level of anxiety (9–15)	40	18.6%
Death anxiety score [Mean (SD)]	7.0 (1.6)	

Moreover, as shown in Table 4, no statistically significant relationship was observed between death anxiety and sociodemographic variables, except monthly income. The participants who had no income had a higher likelihood of developing death anxiety (80%, *p* = 0.016), as shown in Table 4.

**Table 4:** Association between death anxiety and study sociodemographic variables as well as onset of breast cancer.

		**Death anxiety**				**Chi-Square**
		≤ **6**		≥**7**		
		***n***	**%**	***n***	**%**	***p-*** **value**
Age (Years)	18–30	12	54.5%	10	45.5%	0.459
	31–40	30	39.5%	46	60.5%	
	41–50	20	37.7%	33	62.3%	
	51+	27	42.9%	36	57.1%	
Place of residence	Village	35	43.8%	45	56.3%	0.900
	Refugee Camp	14	40.0%	21	60.0%	
	City	40	40.4%	59	59.6%	
Social status	Single	8	57.1%	6	42.9%	0.551
	Married	63	42.3%	86	57.7%	
	Divorced	4	30.8%	9	69.2%	
	Widow	14	36.8%	24	63.2%	
Educational level	Illiterate	18	56.2%	14	43.8%	0.150
	Elementary (1–6 years)	13	43.3%	17	56.7%	
	Preparatory (6–9 years)	12	36.4%	21	63.6%	
	Secondary (10–12 years)	27	47.4%	30	52.6%	
	University	20	32.3%	42	67.7%	
Monthly income	No income	3	20 %	12	80%	0.016
	Less than 300 US$	9	47.4%	10	52.6%	
	301 US$-600 US$	24	64.9%	13	35.1%	
	601 US$-900 US$	38	38.4%	61	61.6%	
	≥ 901 US$	15	34.1%	29	65.9%	
**Onset of breast cancer**	Less than a year	29	37.7%	48	62.3%	0.181
	1–2 years	38	51.4%	36	48.6%	
	3–5 years	11	30.0%	21	70%	
	6 years +	13	41.9%	18	58.1%	

Table 5 presents a significant relationship between death anxiety and the presence of psychological problems such as depression and anxiety (71.2%, *p* = 0.010).

**Table 5:** Association between death anxiety and psychological variables.

		**Death anxiety**				**Chi-square**
		**Less than or equal to 6**		**Equal to or more than 7**		
		***n***	**%**	***n***	**%**	***p*** **-value**
Did you suffer from any psychological problems such as depression and anxiety?	Yes	19	28.8%	47	71.2%	0.010
	No	70	47.3%	78	52.7%	
Did you seek any psychological treatment?	Yes	7	38.9%	11	61.1%	0.745
	No	81	41.3%	115	58.7%	
Do you receive any social support?	Yes	81	42.6%	109	57.4%	0.079
	No	6	25.0%	18	75.0%	

### 3.1 Relationship between the brief COPE scale and templer DAS

Finally, the results of Pearson's correlation test employed to examine the relationships between the application of coping mechanisms and death anxiety are presented in Table 6. The results indicate significant weak and negative correlations between death anxiety and self-distraction (*r* = −0.255, *p* < 0.000), active coping (*r* = −0.200, *p* < 0.003), use of instrumental support (*r* = −0.169, *p* < 0.014), use of emotional support (*r* = −0.162, *p* < 0.018), humor (*r* = −0.293, *p* < 0.000), and acceptance (*r* = −0.147, *p* < 0.033). Furthermore, statistically significant weak and positive correlations were observed between death anxiety and behavioral disengagement (*r* = 0.344, *p* < 0.000), religion (*r* = 0.291, *p* < 0.000), and denial (*r* = 0.258, *p* < 0.000).

**Table 6:** Correlation between coping strategies and death anxiety.

**Coping mechanism**	**Correlation with death anxiety**	***p*-value**
Self-distraction	−0.255^*^	0.000
Active coping	−0.200^*^	0.003
Denial	0.258^*^	0.000
Substance use	0.011	0.876
Use of emotional support	−0.162^*^	0.018
Use of instrumental support	−0.169^*^	0.014
Behavioral disengagement	0.344^*^	0.000
Venting	0.111	0.108
Positive reframing	0.021	0.758
Planning	0.041	0.549
Humor	−0.293^*^	0.000
Acceptance	−0.147^*^	0.033
Religion	0.291^*^	0.000
Self-blame	0.082	0.234

^*^p < 0.05.

### 3.2 multivariate logistic regression for determinants of death anxiety

Table 7 shows the factors that may contribute to the development of death anxiety. Individuals who used positive reframing (AOR = 1.487, CI: 1.049–2.107, *p* < 0.026), self-blame (AOR = 1.309, CI: 1.039–1.650, *p* < 0.023), and religion (AOR = 1.260, CI: 1.021–1.556, *p* < 0.031) as coping mechanisms were more likely to experience death anxiety than those who did not use these strategies. Conversely, individuals who used substances (AOR = 0.657, CI: 0.490–0.879; *p* < 0.005) and active coping (AOR = 0.629, CI: 0.450–0.880, *p* < 0.007) as coping strategies had lower likelihood of experiencing death anxiety.

**Table 7:** Multivariate logistic regression model for the associations between death anxiety and demographic factors, psychological factors, and coping strategies.

	**Death anxiety**		**Adjusted multivariate analysis**	
			**95% CI, AOR** ^*^	
	**p-value**	**AOR**	**Lower**	**Upper**
Active coping	0.007	0.629	0.450	0.880
Positive reframing	0.026	1.487	1.049	2.107
Religion	0.031	1.260	1.021	1.556
Self-blame	0.023	1.309	1.039	1.650
Substance use	0.005	0.657	0.490	0.879

Multivariate logistic regression model: Adjusted for demographic factors including age, place of residence, social status, monthly income, education level, onset of cancer disease; psychological factors including did you suffer from any psychological problem, did you seek any psychological treatment, and do you receive any psychological support; and coping strategies including self-distraction, active coping, denial, substance use, use of emotional support, use of instrumental support, behavioral disengagement, venting, positive reframing, planning, humor, acceptance, religion, and self-blame.

## 4 Discussion

This study aimed to assess the prevalence of death anxiety and its relationship with coping strategies in Palestinian patients with breast cancer. The findings of this study revealed that the majority of participants (58.4%) had symptoms of death anxiety (exhibiting average to high levels of anxiety about death), with a moderate overall mean score for death anxiety of 7.0. In China, Gong et al. (38) found that women with breast cancer had an average death anxiety score of 7.72, while Salehi et al. (39) found that patients had an average death anxiety score of 9.71 ± 3.58, which was higher than the average DAS score for the general population (2.77) (7). In addition, 72.9% of patients with breast cancer reported high levels of death anxiety. According to Shakeri et al. (3), 67.5% of patients with breast cancer experienced death anxiety =. Another study conducted in Iran by Masror Roudsary et al. (9) revealed that 79% of patients with breast cancer experienced death anxiety. Furthermore, a cross-sectional study conducted on 80 breast cancer patients in Pakistan revealed that 51.2% had a medium level of anxiety about death, 27.5% had a high level of anxiety, and 7.5% had an extremely high level of anxiety (10). However, in contrast to the current study, Shakeri et al. (3) reported a prevalence of death anxiety in 67.5% of the breast cancer patients. Furthermore, Soleimani et al.'s (8) systematic review and meta-analysis revealed that death anxiety was only mild in cancer patients (6.84).

Palestinian breast cancer patients may experience high levels of death anxiety related to factors including disease progression, apprehension about their future, and becoming dependent and unable to perform daily tasks (3). Okaye et al. (40) found that oncology patients were concerned about treatment uncertainty. Death anxiety can exacerbate fatigue, sleep disturbances, anxiety, depression, and maladaptation. Individuals who suffer from death anxiety may experience distressing thoughts and difficulties in recovering. Thus, patients require psychological support to cope with death and overcome their fear (8). Palestinian cancer patients do not have access to death anxiety-relieving interventions due to a lack of medical resources and awareness. A robust death education program in Palestinian oncology hospitals and clinics can help breast cancer patients live in the present moment and meaningfully. Effective psychotherapies can help patients deal with death and fear (8).

There have been a few studies that examine the relationship between death anxiety and coping strategies in women with breast cancer (8, 41).

The current study found that participants who used positive reframing, self-blame, or religion as coping strategies were more likely to have death anxiety. By contrast, Bovero et al. (42) found that cancer patients who used coping strategies such as religion and positive reframing were more resilient in the face of death (42). These strategies helped them relieve emotional distress or alter their perception of their medical condition (43). In addition, Guo et al. (44) found that cancer patients who engaged in self-blame experienced higher levels of anxiety.

Self-blame is a maladaptive self-esteem response that involves assigning negative events to one's stable aspects, such as low ability and personal deficiencies. For individuals with a perceived incurable and life-threatening illness, avoiding self-blame as a coping mechanism is crucial. If a person believes they are responsible for their serious illness and attributes it to internal factors, they are likely to feel increased distress, guilt, and low self-esteem. This mindset can make coping with the illness even more difficult because minimizing the psychological impact of the disease is critical for long-term survival (45, 46).

In the current study, using religion as a coping mechanism increased the likelihood of experiencing death anxiety, which was unexpected. Zarzycka and Zietek (47) found that religious people were more distressed and dissatisfied with life. The lack of presence of God in their coping mechanisms resulted in cognitive dissonance (48). Furthermore, distressed cancer patients frequently blame God, causing intense rage toward God and potentially increasing their death anxiety (45). Other studies, however, found a negative correlation between religion and death anxiety (49). Many other studies in the literature review (17, 50) focused on religion's role in reducing death anxiety in breast cancer patients. Elsheshtawy et al. (41) showed that “leaving it in God's hands” helped women cope with uncertainty, illness, and death. The participants believed that God had predestined their path. One possible explanation for Palestinian women with breast cancer having higher levels of death anxiety is that they seek relief through religion but are not intrinsically religious. It has been reported that individuals with an intrinsic religious motivation (who practice their religion) have significantly lower levels of fear of death than those with an external religious motivation (who use religion for social benefits) (51). As a result, hospital and palliative counselors who work with breast cancer patients are recommended to conduct spiritual assessments that include questions about God, the afterlife, and death, as well as offer religious services to these women, to reduce death anxiety.

Furthermore, the current study found that patients who used substances and engaged in active coping strategies were less likely to experience death anxiety. Problem-focused coping involves actions aimed at resolving a problem or situation to improve adaptive adjustment, such as active coping (52). Sharma et al. (53) found that positive problem-solving is the most common coping strategy among breast cancer patients (54). Active coping strategies and problem-solving have been linked to higher moods and enhanced psychological wellbeing. In addition, avoidant coping strategies such as substance use are not always maladaptive and can have varying effects depending on the severity of a patient's symptoms and stress levels (55). Furthermore, research indicates that certain substances, such as psychedelics, may reduce death anxiety (56). However, Krasne et al. (57) reported that cancer patients who relied moderately or heavily on alcohol or drug use as a coping mechanism were more likely to experience anxiety. One explanation for this finding is that women diagnosed with breast cancer often utilize medications such as morphine, codeine, benzodiazepines, Xanax, and Valium to alleviate the side effects associated with cancer or its treatment, including pain, anxiety, and insomnia. These medications can enhance their comfort and alleviate anxiety symptoms, including death anxiety (58).

This study has certain limitations. The cross-sectional designs and convenience sampling make it difficult to establish causality. In addition, the prevalence of death anxiety was assessed using the self-reported DAS, which is not a diagnostic tool. Moreover, various cancer-related variables such as recurrence or metastasis were not evaluated. The study's focus on only Beit Jala Governmental Hospital in Bethlehem implies that the results may not be generalizable to other public or private oncology clinics and centers. Furthermore, almost seven years have passed since data collection; therefore, updated studies on death anxiety among Palestinian women with breast cancer are required. Despite these limitations, this study is the first to assess death anxiety in Palestinian breast cancer patients, making a significant contribution to the literature.

## 5 Conclusion

To address death anxiety among patients diagnosed with breast cancer in Palestinian hospitals, it is crucial to establish and improve mental health services. This should include psychological interventions and counseling provided by mental health professionals in oncology departments. The timely identification of patients facing difficulty in coping is critical for improving distress management and treatment adherence. A psychiatric evaluation should be performed to assess death anxiety in breast cancer patients. This assessment tool enables healthcare professionals to gain a more comprehensive understanding of the coping mechanisms required to meet the psychosocial needs of women with breast cancer.

## Data availability statement

The original contributions presented in the study are included in the article/supplementary material, further inquiries can be directed to the corresponding author.

## Ethics statement

The studies involving humans were approved by the Faculty of Public Health/Mental Health Research Ethics Committee/Al Quds University. The studies were conducted in accordance with the local legislation and institutional requirements. The participants provided their written informed consent to participate in this study.

## Author contributions

MA: Conceptualization, Formal analysis, Investigation, Methodology, Project administration, Software, Supervision, Validation, Writing – original draft, Writing – review & editing. FS: Conceptualization, Data curation, Investigation, Methodology, Validation, Formal analysis, Writing – review & editing. IA: Formal analysis, Writing – review & editing.
